# Changes in the genomes and methylomes of three *Salmonella enterica* serovars after long-term storage in ground black pepper

**DOI:** 10.3389/fmicb.2022.970135

**Published:** 2022-09-09

**Authors:** Cary P. Davies, Thomas Jurkiw, Julie Haendiges, Elizabeth Reed, Nathan Anderson, Elizabeth Grasso-Kelley, Maria Hoffmann, Jie Zheng

**Affiliations:** ^1^Animal Biosciences and Biotechnology Laboratory, Beltsville Agricultural Research Center, NEA, U.S. Department of Agriculture, Beltsville, MD, United States; ^2^Center for Food Safety and Applied Nutrition, Food and Drug Administration, College Park, MD, United States; ^3^Center for Food Safety and Applied Nutrition, Food and Drug Administration, Bedford Park, IL, United States

**Keywords:** *Salmonella*, SNP, methylome, low moisture food, food safety

## Abstract

Low moisture foods (LMFs) have traditionally been recognized as safe for consumption, as most bacteria require higher water content to grow. However, outbreaks due to LMF foods are increasing, and the microbial pathogen *Salmonella enterica* is frequently implicated. *S. enterica* can survive in LMFs for years, but few serovars have been studied, and the mechanisms which underlie this longevity are not well understood. Here, we determine that *S. enterica* serovars *S*. Tennessee, *S*. Anatum, and *S.* Reading but not *S*. Oranienburg can survive in the ground black pepper for 6 years. *S.* Reading was not previously associated with any LMF. Using both Illumina and Pacific Biosciences sequencing technologies, we also document changes in the genomes and methylomes of the surviving serovars over this 6-year period. The three serovars acquired a small number of single nucleotide polymorphisms (SNPs) including seven substitutions (four synonymous, two non-synonymous, and one substitution in a non-coding region), and two insertion-deletions. Nine distinct N6-methyladenine (m6A) methylated motifs across the three serovars were identified including five which were previously known, G^*m*6^ATC, CAG^*m*6^AG, BATGC^*m*6^AT, CRT^*m*6^AYN^6^CTC, and CC^*m*6^AN^7^TGAG, and four novel serovar-specific motifs, GRT^*m*6^AN^8^TTYG, GA^*m*6^ACN^7^GTA, GAA *m*^6*A*^CY, and CAA^*m*6^ANCC. Interestingly, the BATGCAT motif was incompletely methylated (35–64% sites across the genome methylated), suggesting a possible role in gene regulation. Furthermore, the number of methylated BATGC^*m*6^AT motifs increased after storage in ground black pepper for 6 years from 475 to 657 (*S*. Tennessee), 366 to 608 (*S*. Anatum), and 525 to 570 (*S.* Reading), thus warranting further study as an adaptive mechanism. This is the first long-term assessment of genomic changes in *S. enterica* in a low moisture environment, and the first study to examine the methylome of any bacteria over a period of years, to our knowledge. These data contribute to our understanding of *S. enterica* survival in LMFs, and coupled with further studies, will provide the information necessary to design effective interventions which reduce *S. enterica* in LMFs and maintain a healthy, safe food supply.

## Introduction

Low moisture foods (LMFs) are naturally low in moisture or are produced from higher moisture foods through drying or dehydration processes (water activity a_*w*_ < 0.85). Cereals, grains, confections (e.g., chocolate), powdered-protein products (e.g., dairy and egg powders), dried fruits and vegetables, honey, spices, seeds, nuts, and nut-based products (e.g., peanut butter) are among LMF products (Podolak et al., 2010; Beuchat et al., 2013; Finn et al., 2013a). In recent years, there has been an increase in the number of foodborne outbreaks associated with microbial contamination of LMFs, and the pathogen *Salmonella enterica* is often implicated (Vij et al., 2006; Scott et al., 2009; Podolak et al., 2010; Beuchat et al., 2013; Dey et al., 2013; Finn et al., 2013a; Van Doren et al., 2013b). Cereals, grains, and dried protein products are most frequently associated with outbreaks, followed by spices and dried herbs (FAO/WHO, 2014).

This surge in outbreaks is in part due to difficulties in controlling pathogen levels in LMFs. Traditional processing methods which effectively reduce pathogen levels in high moisture foods do not reduce microbial contamination by significant margins (e.g., >5 logs) or to non-detectable levels in LMFs (Beuchat et al., 2013; Finn et al., 2013a). For example, spices and seasonings are often treated with ethylene oxide, propylene oxide, steam treatment, or irradiation to reduce the risk of microbial contamination (Van Doren et al., 2013a), but modifications to these methods are sometimes needed to further reduce levels of *S. enterica* (Newkirk et al., 2018). Contamination may also occur during the production and processing stages, even after a lethal processing step. Furthermore, *Salmonella* strains isolated from low a_*w*_ environments are more likely to acquire tolerance to multiple stresses such as heat, bile salts, and sanitizers than those isolated from high moisture foods (Gruzdev et al., 2011; Smith et al., 2016). Thus, inhibiting the growth of *S. enterica* in LMFs is a continuing challenge.

Survival of *Salmonella* in LMFs for periods of multiple years is well documented (Kotzekidou, 1998; Burnett et al., 2000; Hiramatsu et al., 2005; Uesugi et al., 2007; Keller et al., 2013; Haendiges et al., 2021). Moreover, comparative genomics of *S. enterica* serovar Agona isolates from recurrent multistate outbreaks associated with cereal between 1998 and 2008 demonstrated that *Salmonella* Agona isolates from both outbreaks only differed by a mean of eight single nucleotide polymorphisms (SNPs), highlighting the persistence of *Salmonella* over time in food processing facilities (Hoffmann et al., 2020). The mechanisms by which *S. enterica* responds to the harsh conditions of these environments, however, are not well understood. Populations of *S. enterica* decrease during the initial desiccation period, after which a subset of cells often enters long-term stationary phase (Finn et al., 2013c). Upon desiccation, *Salmonella* undergoes multiple physiological processes (Finn et al., 2013a). Solutes such as trehalose accumulate to regulate cell osmolarity (Csonka, 1989; Csonka and Hanson, 1991), filaments may form (Mattick et al., 2000), and changes occur in cell membrane composition and structure (Garmiri et al., 2008; Finn et al., 2013b). Transcriptomic studies yield mechanistic insights into these processes as genes involved in the heat and cold shock response, DNA protection, and gene regulation among others are either up- or downregulated (Deng et al., 2012; Finn et al., 2013a; Crucello et al., 2019).

Less information is available regarding the physiological events and associated mechanisms during long-term survival of *S. enterica* in low moisture environments. Long-term transcriptional studies have not been performed, and it is unclear whether transcription remains dynamic over a multi-year period after cells enter long-term stationary phase or dormancy. Gene acquisition and loss may affect long-term survival. One study leveraging mutagenesis and sequencing found that genes involved in DNA recombination and repair, osmolarity regulation, lipopolysaccharide biogenesis, stringent response, and stress-induced sigma factors play a role in the survival of *S. enterica* in pistachio, a low a_*w*_ food (Jayeola et al., 2020). In addition, increased rates of DNA mutation of up to ∼10^–10^ mutations per bp per generation can occur as a stress response and some mutations may confer a selective advantage (Bjedov et al., 2003; Foster, 2007). The methyl-directed mismatch repair system or error-prone DNA polymerases may have a role in the generation of this genetic diversity (Goodman, 2002; Foster, 2007). It is therefore possible that mutations which arise and persist in *Salmonella* populations in LMFs may contribute to desiccation tolerance. However, few studies have examined genome-wide mutations in *S. enterica* under conditions associated with food production and processing (Knöppel et al., 2018; Baert et al., 2021), and these were not long-term studies. Data spanning extended periods of time are necessary to understand the mechanisms by which *S. enterica* persists in the food environment and are also critical in the interpretation of data from food safety outbreak traceback investigations such as that of Hoffmann et al. (2020) described above. Such investigations rely on SNP-based phylogenetic analyses to determine isolate relatedness (Allard et al., 2012), and interpretation of these data are contingent on an understanding of genomic changes, particularly over long time periods.

In a previous study, the growth and survival of four *S. enterica* serovars was studied during storage in ground black pepper for 8 months (Keller et al., 2013). Here, the duration of the storage of ground black pepper and paprika contaminated with the same isolates was extended to a period of 6 years to mimic some of the extreme conditions seen in past recurrent outbreaks with LMF. Genomes and DNA methylation patterns of the isolates were compared before and after the long-term storage study. Desiccation tolerance is achieved through multiple adaptive processes (Finn et al., 2013a; Lebre et al., 2017). These genomic and methylomic data will therefore complement existing physiological and transcriptomic approaches by providing a new perspective on the molecular mechanisms which underpin survival processes of *Salmonella* in LMFs.

## Materials and methods

### Bacterial culture

Four *S. enterica* subsp. *enterica* serovars were previously obtained from L. Beuchat, University of Georgia, and stored as stock cultures in tryptic soy broth (TSB) (BD Difco, Becton, Dickinson and Company, Sparks, MD, United States) containing 25% glycerol at −80°C in culture collection of the Division of Microbiology, Center for Food Safety and Applied Nutrition (CFSAN), U.S. Food and Drug Administration (U.S. FDA, College Park, MD, United States). All the isolates including *S*. Tennessee, *S*. Anatum, *S.* Reading, and *S*. Oranienburg were associated with LMFs (Table 1). CFSAN076210 was isolated from a patient in a 2006 peanut associated outbreak in the United States, CFSAN076215 was isolated from raw peanuts in the United States, CFSAN076211 was isolated from raw pecans in the United States, and CFSAN080607 was isolated in the United States (no additional information available). Cultures were plated onto trypticase soy agar with 0.6% yeast (TSAYE, Becton, Dickinson and Company, Sparks, MD, United States) added. Inoculum were prepared as previously described (Keller et al., 2013).

**TABLE 1:** List of isolates used in this study.

Isolates	Serovar	Genbank accession (PacBio)	SRA accession	PacBio RSII	Mi-Seq	Pre or post-pepper	Isolation source	Sequenced bases (Illum/PacBio)
CFSAN076210	Tennessee	CP033345.1, CP033346.1	SRX12580402	x	x	Pre	United States, patient in 2006 peanut associated outbreak	408.7M/4.1G
CFSAN076215	Anatum	CP033338.1, CP033339.1	SRX12580365	x	x	Pre	United States, raw peanuts	495.6M/4.3G
CFSAN080607	Reading	CP068783.1	SRX12580364	x	x	Pre	United States (no additional information available)	458.2M/4.2G
CFSAN076211	Oranienburg	CP033344.1	SRX12580392	x	x	Pre	United States, raw pecans	339.6M/3.9G
CFSAN076147	Reading	CP068788.1	SRX12580390	x	x	Post		471.1M/4.8G
CFSAN076155	Tennessee	CP068786.1, CP068787.1	SRX12580397	x	x	Post		419.6M/2.2G
CFSAN076166	Anatum	CP068784.1, CP068785.1	SRX12580393	x	x	Post		388.4M/2.9G
CFSAN076172	Tennessee		SRX12580363		x	Post		484.2M
CFSAN076163	Anatum		SRX12580396		x	Post		396M
CFSAN076165	Reading		SRX12580391		x	Post		406M
CFSAN076168	Anatum		SRX12580394		x	Post		500.8M
CFSAN076172	Reading		SRX12580395		x	Post		484.2M

### *Salmonella* inoculated spice samples

*Salmonella* inoculated in ground black pepper and paprika samples from a previous study (Keller et al., 2013) were used in this study. In brief, samples were prepared at the Moffett campus, CFSAN, U.S. FDA in 2011. Ground black pepper and paprika were obtained from commercial sources (Keller et al., 2013). Briefly, 25 g quantities of ground black pepper and paprika (not sterilized) were placed into sterile Whirl-Pak filter™ bags (Fisher Scientific, Pittsburgh, PA, United States) and inoculated with 100 μl of undiluted cocktail mix prepared as described earlier to achieve a starting concentration of 10^10^ CFU/g. Inoculum and ground black pepper/paprika were thoroughly mixed and then stored at 25°C at ambient (low) humidity. The a_*w*_ of all 25 g samples was 0.5572 ± 0.0432 at the start of the storage period and was measured using an AquaLab dew point water activity meter model 4TE (Meter Foods, Pullman, WA, United States). During storage, Whirlpak bags were left open in a temperature and relative humidity (RH) controlled incubator equipped with a temperature and RH logger and an emergency power source. Reports indicated that no catastrophic events occurred over the duration of the study. After 6 years of storage, these *Salmonella* inoculated spice samples were shipped to the Division of Microbiology, CFSAN, U.S. FDA for further analysis.

### Recovery of *Salmonella* from ground black pepper and paprika samples

The weight of each sample with the sterile Whirl-Pak bag was recorded. A volume of universal preenrichment broth was added to samples at a 1:9 sample-to-broth ratio and incubated at 35 ± 2°C for 24 ± 2 h. The BAM *Salmonella* culture method for high microbial load foods was followed thereafter (Wallace et al., 2022). Briefly, aliquots of 1.0 and 0.1 ml from the incubated pre-enrichments were subcultured to 10 ml tetrathionate (TT) broth (Becton, Dickinson and Company, Sparks, MD, United States) and to 10 ml Rappaport-Vassiliadis (RV) medium, respectively. TT broth was incubated for 24 ± 2 h at 43 ± 0.2°C, and RV medium for 24 ± 2 h at 42 ± 0.2°C. Each incubated selective enrichment broth was streaked to bismuth sulfite (BS; Becton, Dickinson and Company, Sparks, MD, United States), Hektoen enteric (Becton, Dickinson and Company, Sparks, MD, United States), and xylose lysine desoxycholate (Becton, Dickinson and Company, Sparks, MD, United States) agar plates, which were incubated for 24 ± 2 h at 35 ± 2°C. Five presumptive positive colonies were transferred to triple sugar iron (TSI; BD, Franklin Lakes, NJ, United States) and lysine iron (LIA; BD, Franklin Lakes, NJ, United States) agar slants and incubated for 24 ± 2 h at 35 ± 2°C. Growth from presumptive-positive TSI slants was confirmed as *Salmonella* with VITEK MS (bioMérieux, Durham, NC, United States).

### Molecular serotyping

Enrichment broth RV and TT from each sample and *Salmonella* positive colonies were serotyped using the Luminex xMAP^®^ *Salmonella* Serotyping Assay (Luminex, Madison, WI, United States) to determine composition of surviving serotypes in spice samples. Briefly, DNA was extracted using Bio-Rad InstaGene matrix (Bio-Rad, Hercules, CA, United States) following the manufacturer’s protocol from 1 ml enrichment broth or 1 ml TSB culture from bacterial colony. The standard protocol for the molecular determination of serotype in *Salmonella* from the Centers for Disease Control and Prevention (CDC) was then followed (Fitzgerald et al., 2007; McQuiston et al., 2011).

### Genome sequencing

Original *Salmonella* strains were grown overnight in TSB (Becton, Dickinson and Company, Sparks, MD, United States) and strains recovered from ground black pepper samples after long-term storage were plated onto TSA (Becton, Dickinson and Company, Sparks, MD, United States) and incubated overnight at 37°C. (No strains were recovered from paprika therefore these were not sequenced.) The genomic DNA was extracted using the QiaCube Instrument with the QIAamp DNA Mini kit from Qiagen (Qiagen, CA, United States). Concentrations of gDNA were obtained using the Qubit High Sensitivity dsDNA Kit (Thermo Fisher Scientific, Waltham, MA, United States). All samples were analyzed at the exponential stage of growth.

Seven strains, including one representative of each of the three surviving serovars sampled before and after storage in ground black pepper, as well as the *S*. Oranienburg pre-pepper strain were sequenced using PacBio technology (Table 1). No post-pepper isolate was available for *S*. Oranienburg, as it did not survive in pepper after 6 years of storage. In brief, DNA was sheared to approximately 10 kb using a Covaris g-TUBE (Covaris, Inc., Woburn, MA, United States). SMRTbell 10 kb template libraries were prepared using DNA Template Prep Kit 2.0 and the Low-Input 10 kb Library Protocol (Pacific Biosciences, Menlo Park, CA, United States). DNA was concentrated, repaired, ligated to hairpin adapters, and purified. Incompletely formed SMRTbell templates were digested with a combination of Exonucleases III and VII. Adapters were annealed, and SMRT sequencing was carried out on the PacBio RS II (Pacific Biosciences, Menlo Park, CA, United States) using standard protocols.

Thirteen isolates were sequenced using Illumina technology, including a single pre-pepper isolate, and three post-pepper isolates per serovar (Table 1). Libraries were constructed using the Nextera DNA Prep Library kit (Illumina Inc., San Diego, CA, United States) following the manufacturer’s protocols (Illumina Inc., San Diego, CA, United States). Paired-end DNA sequencing was performed on the Illumina MiSeq using v2 500-cycle sequencing chemistry (2 × 250 bp) (Illumina, Inc., San Diego, CA, United States). Coverage was at least 50 × for all isolates.

### Read processing and genome assembly

Analysis of PacBio sequence reads was implemented using SMRT Analysis 2.3.0 and the SMRT Portal 2.0 platform (Pacific Biosciences; Menlo Park, CA). *De novo* assembly was performed using the Hierarchical Genome Assembly Process (HGAP) with default parameters (Bjedov et al., 2003), as described previously (Pirone-Davies et al., 2015). Genomes were annotated using the NCBI (National Center for Biotechnology Information) Prokaryotic Genomes Automatic Annotation Pipeline (Goodman, 2002).^1^ Adapters were removed from MiSeq reads using bcl2fastq (Illumina, 2017) and reads were trimmed to phred 20 with Trimmomatic v. 0.36 (Bolger et al., 2014), assembled with SPAdes v3.13.0 (Bankevich et al., 2012), and annotated with Prokka (Seemann, 2014).

### Detection of single nucleotide polymorphisms, indels, and genes in pre- and post-pepper isolates

Single nucleotide polymorphismSNP differences between pre- and post-pepper isolates were identified using the CFSAN-SNP pipeline v. 2.2.0 (Davis et al., 2015) with default parameters. This pipeline maps Illumina reads to a reference genome with Bowtie2, processes the mapping (BAM) files using SAMtools, and identifies variant sites using VarScan. A separate analysis was performed for each serovar, with MiSeq reads as input, and the PacBio pre-pepper assemblies used as the reference genomes (Tennessee, CFSAN076210; Anatum, CFSAN076215; and Reading, CFSAN080607). To verify results of the CFSAN Pipeline, and also to detect single nucleotide insertion-deletions (indels) in post-pepper isolates, the SNP detection workflow of mummer v. 3.23 (Kurtz et al., 2004) was used with default parameters to compare pre- and post-pepper genome assemblies. As with the CFSAN pipeline analyses, the PacBio pre-pepper assemblies were used as the pre-pepper reference genomes, and MiSeq assemblies were used to represent each post-pepper isolate. Last, mummer was also used to compare the PacBio pre- and post-pepper assemblies. However, no new SNPs were identified using this method. Furthermore, indels identified in homopolymeric stretches could not be distinguished from sequencing errors as PacBio sequences are prone to errors in these regions. Thus, we do not report results from these analyses. In addition, a gene presence-absence analysis was conducted on isolates from each serovar using Roary 3.10.2 (Page et al., 2015). Sequences identified as present in some, but not all isolates, were manually checked to ensure sequences were not split between two contigs, and positioned at contig edges. If they were, we assumed these homologs were truncated in the annotation protein fasta and were likely excluded during the filtering step of Roary which removes partial protein sequences. We therefore did not count these as differentially identified among isolates. In other cases, homologs had been classified into distinct Roary clusters, even when sharing high sequence similarity (pident ≥ 95%, qcov ≥ 95%). These sequences were therefore added into the appropriate existing clusters.

### Methylation analysis

Motif Detection and Analysis was also carried out using SMRT Analysis 1.1 and the RS_Modification_and_Motif_Analysis.1 protocol as described at https://www.pacb.com/wp-content/uploads/2015/09/WP_Detecting_DNA_Base_Modifications_Using_SMRT_Sequencing.pdf. Interpulse durations (IPDs) were measured based on the kinetic signals (Flusberg et al., 2010) and processed as described previously (Clark et al., 2012). IPD is the amount of time between emission pulses indicative of base incorporation events during sequencing, and is used to determine the methylation status of a base. At each position in the genome, the observed IPD was compared to the IPD of an *in silico* control using a two-sample *t*-test, and a QV score was calculated as QV = −10 log (*p*-value). Bases were accepted as modified based on a minimum QV threshold value. QV 30 was used as a threshold for preliminary analyses. A plot of QV versus coverage was then constructed using publicly available R scripts found at: https://github.com/topics/motif-finding. The observed bimodal distribution of kinetic data, resulting from modified and unmodified positions, was then used to determine a more stringent QV threshold. Only sites with a minimum of 25× coverage were included. Motifs were identified using the algorithm MotifMaker.^2^ m6A and m4C motifs can be reliably detected with 25× coverage across all positions in the genome, but m5C requires either significantly higher coverage (∼250×) or Tet-methylation for confident detection. In this study, we consider only m6A and m4C methylations.

Custom python scripts were used to determine whether methylated BATGCAT motifs were present in coding sequence regions (CDSs) or non-coding regions (between CDSs), with a CDS region defined by the biopython packages Bio.SeqIO and Bio.SeqFeature with feature = CDS (Cock et al., 2009; Mckinney, 2010). Sequences of methylations present in coding and non-coding regions of pre- and post-pepper isolates were compared using blastn (Altschul et al., 1990) (pident ≤ 95, qcov ≤ 95). Sequences of methylated coding and non-coding regions were compared across the three serovars using cd-hit (Li and Godzik, 2006), with sequences clustered at 95% identity. Methylated BATGCAT motifs present on plasmids were not considered for these comparisons, as there was a high proportion of sites in the plasmids which did not reach the minimum of 25× coverage required for accurate signal detection.

## Results

### Recovery of *Salmonella enterica* serovars in ground black pepper after long-term storage

*Salmonella* was recovered from all five ground black pepper samples, while none of the paprika samples had a detectable level of *Salmonella*. Among *Salmonella*-positive ground black pepper samples, serotypes Reading and Anatum were recovered from all RV and TT samples. Serotype Tennessee was recovered from four of the ground black pepper samples. It was noted that more TT samples were found to have serotype Tennessee than RV samples. None of the samples were observed to contain serotype Oranienburg with both culture method and direct molecular serotyping from enrichment broth.

### Comparative genomics

Few SNP differences between pre- and post-pepper isolates were observed. In *S.* Tennessee, the CFSAN-SNP pipeline reported the presence of one substitution shared by CFSAN076155 and CFSAN076150, and one additional substitution present in CFSAN076155. In *S.* Anatum, a single substitution was observed in the three post-pepper isolates, each of which was unique. Of the four *S.* Reading post-pepper isolates, two isolates, CFSAN076165 and CFSAN076177, each contained a unique SNP substitution (Table 2).

**TABLE 2:** Single nucleotide polymorphisms identified across all *S. enterica* isolates.

Post-pepper isolate (serovar)	Pre-pepper isolate position	Nucleotide change	Gene	Locus tag	Functional annotation of SNP position	Detect by CFSAN SNP pipeline	Detect by mummer SNP pipeline	(Non) synonymous (syn)/(non-syn)/frameshift
CFSAN076150, CFSAN076155 (Tennessee)	CFSAN076210 225520	G to A	eno	EBE17_RS04970	Phosphor-pyruvate hydratase, eno = enolase	Yes	Yes	Syn
CFSAN076150, CFSAN076155 (Tennessee)	CFSAN0762101010867	A to “–”	MazG	EBE17_05030	Nucleoside triphosphate pyrophosphohydrolase	No	Yes	Frameshift
CFSAN076150, CFSAN076155 (Tennessee)	CFSAN0762104769694	T to C	ilvL/no gene name	EBE17_24050/EBE17_24055	Intergenic: IlvGMEDA operon leader peptide EBE17_24050 and ATP-dependent protease EBE17_24055	Yes	Yes	NA in non-coding region
CFSAN076163 (Anatum)	CFSAN0762152201889	C to T	EAE34_11170	EAE34_11170	methylated-DNA–[protein]-cysteine S-methyltransferase	Yes	Yes	Non-syn
CFSAN076168 (Anatum)	CFSAN0762153388361	A to G	EAE34_17290	EAE34_17290	EAL domain-containing protein	Yes	Yes	Syn
CFSAN076166 (Anatum)	CFSAN0762154568091	A to G	EAE34_23050	EAE34_23050	Homoserine/homoserine lactone efflux protein	Yes	Yes	Syn
CFSAN076163, CFSAN076166, CFSAN076168 (Anatum)	CFSAN076215668784	“–” to A	EAE34_03275	EAE34_03275	Flotillin family protein	No	Yes	Frameshift
CFSAN076165 (Reading)	CFSAN0806071293683	A to G	dmsA_1	HOMILEEO_01227	Dimethyl sulfoxide reductase DmsA	Yes	Yes	Syn
CFSAN076177 (Reading)	CFSAN0806073291057	T to G	fepE	HOMILEEO_03178	Ferric enterobactin transport protein FepE	Yes	Yes	Non-syn

The mummer pipeline identified all SNPs identified in the CFSAN pipeline, as well as several single nucleotide insertion-deletions (indels). All indels were present in homopolymeric stretches of cytosines or guanines. After manual examination of MiSeq reads from all pre- and post-pepper isolates against the PacBio reference pre-pepper genomes, most indels were deemed to be the result of PacBio sequencing errors in homopolymeric stretches, except for two indels in serovars *S*. Tennessee and *S*. Anatum.

No differences in gene presence-absence were observed between the pre- and post-pepper isolates in any serovar. Roary identified sequences which were present in only a subset of the isolates examined. However, after blastn analyses, it was determined that homologs of the sequences were present in all genomes (pident ≥ 95%, qcov ≥ 95%).

### Comparative methylomics

**FIGURE 1:**
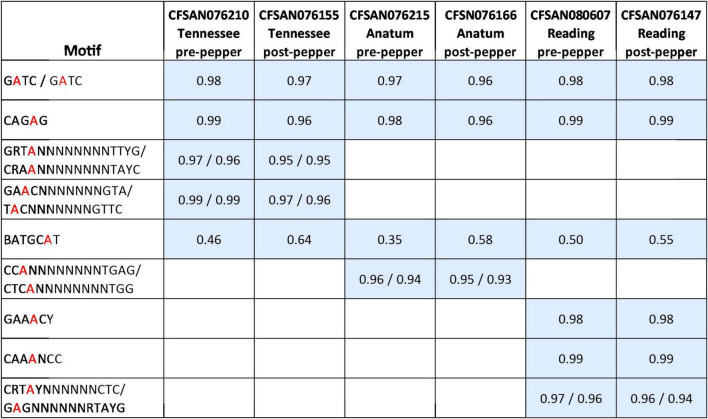
Methylated motifs identified in pre- and post-pepper isolates of three *S. enterica* serovars, with the percentage of methylation of each motif shown in gray squares (number of methylated motifs/total number of motifs present in genome). BATGCAT is the only motif which is partially methylated (i.e., fewer than 95% of sites are methylated throughout the genome).

We observed nine distinct N^6^-methyladenine (m6A) methylated motifs across the three serovars, with a total of five in *S*. Tennessee, four in *S*. Anatum, and six in *S.* Reading (Figure 1). The motifs G^m6^ATC, CAG^m6^AG, and BATGC^m6^AT or BATGC^m6^ATV (B = C,G, or T; V = A,C, or G) were identified in all serovars (Figure 1). In *S*. Tennessee, GRT ^m6^AN_8_TTYG, and GA^m6^ACN_7_GTA, were also identified. In *S*. Anatum, CC^m6^AN_8_TGAG was observed, and in *S.* Reading, CRT^m6^AYN_6_CTC, GAA ^m6^ACY, and CAA ^m6^ANCC were identified. At least 93% of all 6mA sites within the identified motifs were methylated throughout the genomes, except for the motif BATGCAT, which was partially methylated in all three serovars (35–64%). In *S*. Anatum, two 4mC motifs, G^m4^CSBYVNBG and G^m4^CGSNNTBH, were detected in the post-pepper isolate CFSAN076166, and a single 4mC motif, G^m4^CGBNDNTB, was detected in the pre-pepper isolate CFSAN076215. However, the mean motif scores for each motif were less than 100, and the percentage of each motif that was methylated throughout the genome was low (6–10%). These motifs were not confidently identified and are thus not included in our final motif list (Figure 1).

In CFSAN076215 (*S*. Anatum, pre-pepper genome) and CFSAN080607 (*S.* Reading, pre-pepper genome), the motif BATGC^m6^ATV (B = C or G or T; V = A or C or G) was identified, while BATGC^m6^AT was identified in the remaining genomes. In all six genomes, the modifications.gff files from the SMRTPortal pipeline were parsed to determine the percentage of methylated ATGC^m6^ATT sites. We found that a similar but slightly lower number of ATGC^m6^ATT sites were methylated in CFSAN076215 and CFSAN080607 (9 and 11%, respectively) compared to the remaining genomes (13–16%), which likely led to the differential reporting of BATGC^m6^ATV and BATGC^m6^AT in the MotifMaker output. The percentages of ATGC^m6^ATT sites methylated across all six genomes do not differ greatly, thus we report on the methylation of BATGC^m6^AT in all genomes for the remainder of the paper, both for the sake of consistency and to be able to compare the frequency of methylation across pre- and post-pepper isolates. Additional work is needed to determine whether these are true biases in the motifs identified.

**FIGURE 2:**
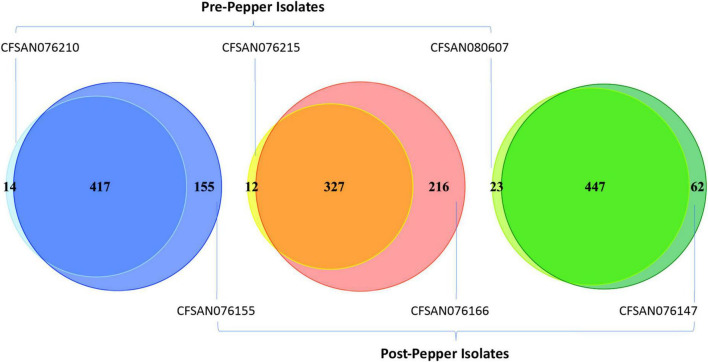
Venn diagrams showing the number of BATGC^*m*6^AT motifs before and after immersion in black pepper for the three serovars (from left to right, Tennessee, Anatum, Reading). Most motifs remain methylated throughout storage in black pepper, and some additional motifs are methylated.

The percentage of each motif that was methylated throughout the genome was similar before and after immersion in black pepper, except for that of BATGC^m6^AT (Figures 1, 2). The total number of methylated BATGC^m6^AT motifs increased after immersion in pepper in all three serovars from 475 to 657 (*S*. Tennessee), 366 to 608 (*S*. Anatum), and 525 to 570 (*S.* Reading). Approximately equal numbers of methylated BATGC^m6^AT motifs were present in coding and non-coding regions across all three serovars (Table 3). For example, the pre-pepper Tennessee genome CFSAN076210 contained a total of 475 BATGC^m6^AT methylations, 231 of which were present within 214 coding regions, and 244 of which occurred in 217 non-coding regions. No methylations of this motif were present in any regions annotated as a non-gene Genbank feature, including different types of RNAs (ncRNA, rRNA, tRNA, and tmRNA), repeat regions, or miscellaneous features. The majority of the methylations in pre-pepper isolates were maintained after immersion in pepper, and additional sites were methylated in each post-pepper genome (Figure 2).

**TABLE 3:** Total number of BATGC^*m*6^AT motifs present in the genomes.

Isolate	Serovar	Pre/Post immersion in pepper	Total # BATGC^*m*6^AT motifs	Total # BATGC^*m*6^AT motifs, coding regions	Total # BATGC^*m*6^AT motifs, non-coding regions
CFSAN076210	Tennessee	Pre	475	231	244
CFSAN076155	Tennessee	Post	657	295	362
CFSAN076215	Anatum	Pre	366	171	195
CFSAN076166	Anatum	Post	608	272	336
CFSAN080607	Reading	Pre	525	238	287
CFSAN076147	Reading	Post	570	256	340

We also assessed whether methylated BATGC^m6^AT motifs occurred in homologous regions across the genomes of the three serovars, with a region defined as the CDS or inter-CDS region in which the methylated adenine was located (see methods for details). Before immersion in pepper, there were 222 methylated regions which were homologous among all three serovars, while after immersion in pepper, this number increased to 377.

## Discussion

Dried ground black pepper is an inhospitable environment for *S. enterica*. Not only are moisture levels low, but multiple antimicrobial compounds are also present, including 1-phellandrene and caryophyllene (Jirovetz et al., 2002). Still, we recovered serovars *S*. Tennessee, *S*. Anatum, and *S.* Reading from dried ground black pepper after 6 years. *S.* Reading was not previously associated with LMFs. These results extend our current knowledge on the longevity of *S. enterica* in low a_*w*_ foods (Kotzekidou, 1998; Burnett et al., 2000; Hiramatsu et al., 2005; Uesugi et al., 2007; Keller et al., 2013). It is noted that serovar Tennessee was recovered more frequently from TT than RV media.

It is unclear why *Salmonella* was not recovered from paprika samples. To determine whether *Salmonella* was present but in a dormant state, we attempted to resuscitate *Salmonella* in nutrient rich medium, TSB, for 20 h. Still, *Salmonella* was not recovered. Given the observed serovar-specific survival of *Salmonella* in black pepper, it is possible that *Salmonella* survival in paprika may also be serovar-specific. *Salmonella* serovars Saint Paul, Javiana, and Rubislaw were isolated from paprika and paprika products associated with an outbreak (Lehmacher et al., 1995), and *S*. Typhimurium in paprika was studied under controlled laboratory conditions (Shankarrao Shirkole et al., 2021). However, the majority of *Salmonella* serovars have not been studied, and it is likely that a differential response to the low moisture and phytochemicals present in paprika could affect survival.

Transcriptome studies of Salmonella in LMFs provide information on the molecular mechanisms underlying short-term responses to low a_*w*_ environments. In peanut oil, only 1.36–4.42% of the *S*. Enteritidis genome was expressed by hours 216 and 528, suggested a significant reduction in overall metabolic activity. Sigma factor RpoE, and cold and heat shock proteins were among the few differentially expressed genes (DEGs) (Deng et al., 2012). In a comparison of *S*. Typhimurium in black pepper, milk chocolate, powdered milk, and dried pet food, few DEGs were reported to be shared at 24 h except for cold shock genes, but this number increased at 72 h, suggesting a convergence of gene expression across all products at this later timepoints (Crucello et al., 2019). In black pepper, DEGs included ribosomal genes, tRNA genes, and plasmid borne genes involved in horizontal gene transfer (Crucello et al., 2019).

Few studies have examined genomic mutations in *Salmonella* in LMFs. Baert et al. (2021) reported on SNPs in *S. enterica* subjected to low a_*w*_ conditions (Knöppel et al., 2018; Baert et al., 2021). *Salmonella* was exposed to 10 cycles of heat treatment while in a low a_*w*_, high fat matrix. Resulting isolates differed by 0–28 SNPs (Baert et al., 2021), but isolates were cultured in between cycles, thus these results may not be representative of genomic changes which occur outside of the laboratory. In a food safety outbreak traceback investigation, Hoffmann et al. (2020) showed that *S*. Agona strains can survive and persist in the processing environment for 10 years with only 6 SNPs difference in average (Hoffmann et al., 2020).

Here, over a period of 6 years, we detected seven substitutions and two indels among the three serovars stored in ground black pepper (Table 2), and four of the mutations occur in genes involved in stress-related processes. Four synonymous mutations were identified across the serovars. Synonymous mutations can alter messenger RNA (mRNA) structure and affect translation efficiency, mRNA stability, or protein folding (Lind et al., 2010). Thus, we predicted the effects of the mutations on mRNA secondary structure using the program RNAsnp.^3^ Of the four mutations, only those found in the enolase (*p*-value = 0.1716) and a homoserine/homoserine lactone efflux protein (*p*-value = 0.0752), were predicted to result in structural disruptions, but the *p*-values were high. We also examined whether the four mutations could affect tRNA usage and ultimately affect translation efficiency (Frumkin et al., 2018; Hanson and Coller, 2018). However, the four mutations are each present in the third codon position and therefore do not likely affect tRNA recognition. It is therefore unlikely that these mutations contribute to *Salmonella* survival in pepper.

Two non-synonymous mutations were detected, one in *S.* Anatum, and one in *S.* Reading. In *S.* Anatum, a cytosine was replaced with a thymine within a gene encoding a methylated-DNA–protein-cysteine methyltransferase, homologous to *ogt*, which is involved in the repair of DNA alkylation damage (Daniels and Tainer, 2000; Pegg, 2000). This mutation results in the translation of a lysine instead of a glutamate and leads to a reversal in charge from negative to positive. According to the crystal structure of a homolog from *Sulfolobus* (Perugino et al., 2015), the mutation occurs in a charged loop region distant from the enzyme active site. DNA damage can be caused in stressed cells due to the accumulation of reactive oxygen species, therefore if this function is enhanced by this mutation, it could confer a fitness benefit in a dry environment. The second non-synonymous mutation occurred in the *fepE* gene of *S.* Reading which encodes for a membrane protein involved in the biosynthesis of long chain O-antigens in lipopolysaccharides (Murray et al., 2003; Morona et al., 2009). The mutation occurs at the end of an alpha helix near the protein-protein interaction surface between *fepE* monomers (Tocilj et al., 2008), and would result in the translation of an arginine in place of a serine. FepE interacts with other Wzz proteins to determine O-antigen length. Efforts to determine the exact mechanism by which this occurs are ongoing, but mutations in multiple sites of *fepE* and other *Wzz* genes can alter O-antigen length (Tran and Morona, 2013; Wiseman et al., 2021). Variable O-antigen length can affect the interactions between *Salmonella*, host, and the environment (Murray Gerald et al., 2006; May and Groisman, 2013), and changes in O-antigen length facilitated by *fepE* contribute to desiccation tolerance in *Cronobacter* (Qian et al., 2021). Interestingly, a mutation in the gene *gtr* which also contributes to O-antigen length arose multiple times when *Salmonella* was repeatedly exposed to heat in a low a_*w*_ high fat matrix (Baert et al., 2021). However, the authors conclude that further studies are needed to determine whether the mutation contributes to fitness under stressful conditions.

A single substitution was detected in a non-coding region of the genomes of two *S.* Tennessee isolates, CFSAN076150 and CFSAN076155. The mutation occurred 75 bases upstream of an ATP-dependent protease, and 277 bases upstream of the IlvGMEDA operon leader peptide. Two single nucleotide indels were observed across all three serovars. In *S.* Anatum, an insertion occurred in a flotillin family protein in all three isolates and would lead to a frameshift resulting in the translation of two small proteins (52 and 506 amino acids) instead of one longer one (559 amino acids). Flotillins are inner membrane proteins which facilitate the formation of membrane domains and alter membrane fluidity, and thus contribute to many physiological processes (Bach and Bramkamp, 2013; Padilla-Vaca et al., 2019). Flotillin has not been previously reported to play a role in desiccation. However, changes in membrane structure and function including increases in membrane fluidity do occur in *Salmonella* upon desiccation (Gruzdev et al., 2012; Lebre et al., 2017), therefore the role of flotillin in the response of *Salmonella* in LMFs should be further investigated.

In *S*. Tennessee, a deletion occurred in *mazG*, a nucleoside triphosphate pyrophosphohydrolase which plays a role in adaptation to stress (Zhang and Inouye, 2002; Gross et al., 2006; Lu et al., 2010). This enzyme can hydrolyze non-canonical nucleotides which may otherwise be erroneously incorporated into DNA and RNA (Lu et al., 2010; Lyu et al., 2013), and it also interferes with the stringent stress response and programmed cell death (PCD) induced by the toxin *mazF* (Gross et al., 2006). The resultant frameshift would lead to a truncation in the *mazG* protein (266 to 210 amino acids), and a loss of one of the two *mazG* domains, the Nucleoside Triphosphate Pyrophosphohydrolase (EC 3.6.1.8) C-terminal tandem-domain (cd11528). In *Escherichia coli*, *mazG* contains both C- and N-terminal tandem domains, but there are other *mazG* homologs which contain only a single domain (Robinson et al., 2007; Lu et al., 2010). Thus, it is possible that this mutation does not result in a loss of gene function. In addition, a second open reading frame (orf) is present in the mutated sequence which, when translated, would be 87 amino acids in length and span a full-length C-terminal *mazG* domain. Either the truncated protein, or the two novel proteins, may remain active, as it would be advantageous to maintain the functioning of this gene in an inhospitable environment such as black pepper.

The documentation of nucleotide mutations in *S. enterica* over a 6-year period in ground black pepper provides a first look at how the genomes of *S. enterica* change over long periods of time in LMFs. Although few mutations were observed, four of nine mutations occurred in genes related to stress responses within the cell, and further studies will determine whether any of the mutations confer a selective advantage to *S. enterica* under stressful conditions. This information will be useful in understanding whether DNA mutations contribute to long-term isolate survival in LMFs, and for the interpretation of data for *S. enterica* food safety outbreak traceback investigations. Such investigations often determine isolate similarity based on the construction of whole genome sequencing (WGS) phylogenies and pairwise calculation of SNP distances between isolates (Allard et al., 2012). In some cases, similarities between isolates collected over periods of multiple years must be determined (Hoffmann et al., 2020). Mutation rates for *S. enterica* in specific environments are useful the interpretation of these studies, but these data are not available for *S. enterica* in LMFs over multi-year periods. Thus, observations regarding the acquisition of mutations among the three serovars over 6 years will aid in these interpretations.

DNA methylation may affect the ability of *S. enterica* to adapt and survive in the black pepper environment. In prokaryotes, methylation of the N-6 position of adenine (6mA) is the most common DNA modification and is achieved by a methyltransferase enzyme (MTase) (Fujimoto et al., 1965; Casadesus and Low, 2006). MTases, along with a cognate restriction endonuclease, often occur as a restriction-modification system (RM system), which protects cells from foreign DNA (Roberts, 2003; Loenen et al., 2013). However, DNA methylation is also involved in diverse cellular functions including DNA replication initiation and DNA repair, virulence, and gene regulation, among others (Low et al., 2001; Wion and Casadesus, 2006; Shell et al., 2013; Adhikari and Curtis, 2016; Beaulaurier et al., 2019). In *S. enterica*, the most well-studied MTase is deoxyadenosine methyltransferase (Dam), which methylates the 5′-GATC-3′ motif (Adhikari and Curtis, 2016). Dam regulates the expression of some virulence genes including the plasmid-encoded fimbriae (pef) locus and the std fimbrial operon in *S. enterica* (Nicolson and Low, 2000; Jakomin et al., 2008). Little is known about the functions of additional methylated motifs in bacteria, and to our knowledge no studies have focused on the function of motifs in *Salmonella* aside from GATC. However, our prior research showed that the motif ATGCAT is partially methylated (Pirone-Davies et al., 2015), which may indicate the involvement of the motif in gene regulation (Lluch-Senar et al., 2013; Blow et al., 2016). Thus, as a pilot study, we documented 6mA motifs in the genomes of one pre- and one post- pepper isolate from each serovar (Table 1) to determine whether changes occur in methylation after immersion in black pepper, as changes may imply a role in the adaption to the black pepper environment, possibly *via* differential gene regulation.

We detected a total of nine methylated motifs in the three *Salmonella* serovars, four of which are not previously described, GRT^m6^AN_8_TTYG, GA^m6^ACN_7_GTA, GAA ^m6^ACY, and CAA ^m6^ANCC. G^m6^ATC, CAG^m6^AG, and BATGC^m6^AT, were methylated across all three serovars, while CRT^m6^AYN_6_CTC, CC^m6^AN_8_TGAG, GRT^m6^AN_8_TTYG, GA^m6^ACN_7_GTA, GAA ^m6^ACY, and CAA ^m6^ANCC were serovar-specific (Figure 1). These results are similar to those of a previous study, which also observed the conservation of the three motifs across serovars, in addition to serovar-specific motifs (Pirone-Davies et al., 2015). Further comparison of data from this study with that of the previous suggests that within-serovar variation of methylation motifs exists. All motifs in the two *S*. Anatum genomes examined here were identical to those previously identified in *S*. Anatum ATCC-BAA1592 (Pirone-Davies et al., 2015), except for a 4mC motif that was identified in *S*. Anatum ATCC-BAA1592, ^m4^CCWWGG, but not in this study. This motif is methylated by a Type II methyltransferase M.SenAnaIV (deposited in the REBASE database (rebase.neb.com); locus tag SEEA1592_11855). We performed a blastn search of this enzyme against the genomes of *S*. Anatum CFSAN076215 and CFSAN076166, and did not find any hits, further suggesting that this motif is not likely methylated across all *S.* Anatum genomes. Previous studies in *Listeria monocytogenes* (Chen et al., 2017), *S. enterica* (Pirone-Davies et al., 2015), and *Bifidobacterium breve* (Bottacini et al., 2018) indicate variations in MTases and methylated motifs occur among species and serovars, but only identical 6mA motifs were identified within different strains of the same serovars (*S*. Enteritidis and *S*. Heidelberg) (Pirone-Davies et al., 2015).

A single motif, BATGC^m6^AT, was incompletely methylated (35–64% of all genome sites methylated), as was found previously (Pirone-Davies et al., 2015). Incomplete methylation may play a role in gene regulation (Lluch-Senar et al., 2013; Blow et al., 2016). Thus, it is possible that methylation of this motif may provide a means for *S. enterica* to adapt to the stressful environment of black pepper, perhaps by altering gene expression. Thus, we further examined patterns of BATGC^m6^AT methylation across the three serovars.

Of the methylated BATGC^m6^AT motifs present in non-coding regions, four occur in promoter regions for genes including the molecular chaperone *dnaJ*, two distinct MFS transporters, and a hypothetical protein. These four motifs were present in all six genomes examined, except CFSAN076215 only contained this motif in one MFS transporter. In addition, the two *S*. Tennessee genomes contained a methylated BATGC^m6^AT in the promoter regions of the genes Ag (+)-translocating P-type ATPase SilP and a multidrug efflux MFS transporter. Transcriptional studies are needed to determine the effect of these and other methylations on gene expression, if any.

Approximately half of all methylated BATGC^m6^AT motifs (50–57%) in pre-pepper genomes occurred in regions homologous across the serovars, and this percentage increased after immersion in pepper [Tennessee, 51–66%, Anatum, 57–69%, and 50–74% (# methylated BATGC^m6^AT motifs located in homologous regions/# total methylated BATGC^m6^AT motifs per genome, before and after immersion in pepper)]. This methylation of BATGC^m6^AT across conserved regions of the serovars suggests a functional role for the methylations.

To our knowledge, this is the first study to characterize long-term changes in the methylome of *Salmonella*. Further studies which characterize the role of the methylation of various motifs in *S. enterica*, particularly of BATGC^m6^AT, in black pepper and other environments are needed. One limitation of this study is the analysis of the post-pepper methylomes after growth in media, instead of directly after immersion in black pepper as growth in broth may alter the methylome. However, pre-pepper isolates were also immersed in broth in an identical procedure for sequencing before storage in pepper, and consistent changes in the methylation of BATGC^m6^AT were still observed across all three serovars. This suggests that methylomic signals due to storage in pepper were detectable.

An increasing number of outbreaks are associated with LMFs contaminated with *S. enterica*. Thus, an enhanced understanding of the mechanisms by which this pathogen adapts to and survives in low moisture environments is needed. In this pilot study, we demonstrate that three *S. enterica* serovars survive in black pepper for 6 years and acquire a small number of single nucleotide mutations. We also show that among all serovars, the number of BATGC^m6^AT sites increased after immersion in pepper, and a high proportion of BATGC^m6^AT sites were present in homologous regions of the genomes from the three serovars. No mutations occurred within methylated motifs. Additional studies which determine the precise mutation rates of *S. enterica* in LMFs and elucidate the role of nucleotide and methylation changes in *S. enterica* physiology are needed. Together, these data will provide the basis for the design of effective intervention strategies which eliminate *S. enterica* from LMFs and safeguard the food supply.

## Data availability statement

The datasets presented in this study can be found in online repositories. The names of the repository/repositories and accession number(s) can be found below: https://www.ncbi.nlm.nih.gov/genbank/, SRX12580390, SRX125803 97, SRX12580393, SRX12580402, SRX12580365, SRX12580364, SRX12580363, SRX12580396, SRX12580391, SRX12580394, SRX12580395, SRX12580410, SRX12580392, CP033338.1, CP0 33339.1, CP033344.1, CP033345.1, CP033346.1, CP068783.1, CP068784.1, CP068785.1, CP068786.1, CP068787.1, and CP068788.1.

## Author contributions

CD, JZ, MH, and TJ: data interpretation. CD, JZ, MH, TJ, NA, and EG-K: conceptualization. CD, JZ, TJ, JH, and MH: formal analysis. TJ, JH, JZ, MH, ER, and NA: data generation. JZ, MH, and CD: supervision. CD, JZ, and TJ: writing original draft. JZ, CD, MH, TJ, JH, NA, and EG-K: writing—review and editing. All authors have read and approved the final manuscript.

## Footnotes

1
http://www.ncbi.nlm.nih.gov/genomes/static/Pipeline.html

2
https://github.com/bioinfomaticsCSU/MultiMotifMaker

3
https://rth.dk/resources/rnasnp/
